# Functional joint regeneration is achieved using reintegration mechanism in *Xenopus laevis*

**DOI:** 10.1002/reg2.49

**Published:** 2016-01-06

**Authors:** Rio Tsutsumi, Shigehito Yamada, Kiyokazu Agata

**Affiliations:** ^1^ Department of Biophysics, Graduate School of ScienceKyoto UniversityKyotoJapan; ^2^ Human Health Science, Graduate School of MedicineKyoto UniversityKyotoJapan; ^3^ Congenital Anomaly Research Center, Graduate School of MedicineKyoto UniversityKyotoJapan

**Keywords:** Frog, joint, limb, regeneration, reintegration

## Abstract

A functional joint requires integration of multiple tissues: the apposing skeletal elements should form an interlocking structure, and muscles should insert into skeletal tissues via tendons across the joint. Whereas newts can regenerate functional joints after amputation, *Xenopus laevis* regenerates a cartilaginous rod without joints, a “spike.” Previously we reported that the reintegration mechanism between the remaining and regenerated tissues has a significant effect on regenerating joint morphogenesis during elbow joint regeneration in newt. Based on this insight into the importance of reintegration, we amputated frogs’ limbs at the elbow joint and found that frogs could regenerate a functional elbow joint between the remaining tissues and regenerated spike. During regeneration, the regenerating cartilage was partially connected to the remaining articular cartilage to reform the interlocking structure of the elbow joint at the proximal end of the spike. Furthermore, the muscles of the remaining part inserted into the regenerated spike cartilage via tendons. This study might open up an avenue for analyzing molecular and cellular mechanisms of joint regeneration using *Xenopus*.

## Introduction

Limb regenerative ability varies among vertebrates (Wallace 1981; Agata & Inoue 2012; Seifert et al. 2012). Mammals, including humans, cannot regenerate the lost structure after amputation proximal to the terminal joint of their digits; they are incapable of regenerating joints (Douglas 1972; Illingworth 1974; Han et al. 2008; Muneoka et al. 2008). In contrast, many urodele amphibians (newts and salamanders) are able to regenerate the complete structure of their limbs after amputation throughout their lives. In anuran amphibians (frogs and toads), perfect limb regeneration is only observed during the larval stage, and they become unable to regenerate the complete structure of the limb after metamorphosis (Dent 1962; Goode 1967). Adult *Xenopus laevis*, for example, regenerate a hypomorphic single cartilage rod called a spike, without joints, muscles, mineralized bones, tendons, or ligaments, after amputation at any level along the limb axis (Endo et al. 2000; Tassava 2004; Satoh et al. 2005a, 2006; Suzuki et al. 2006). Thus, adult frog limb regeneration is regarded as an intermediate model between urodele amphibian and mammalian limb regeneration (Seifert et al. 2012).

In both urodele and anuran amphibians, after a limb is amputated, wound epithelium soon covers the wound surface, and the cells in the stump tissue lose their differentiated‐cell features and start to proliferate underneath the wound epithelium; as a result, a blastema is formed (Christensen & Tassava 2000; Christensen et al. 2002; Agata et al. 2007). After blastema formation, the blastema epimorphically and autonomously redevelops the distal structure. In addition, there is a process by which the epimorphically regenerated tissues and the original tissues are reintegrated, which is called the reintegration mechanism (Carlson 2007; Makanae et al. 2014; McCusker & Gardiner 2014; Tsutsumi et al. 2015). In urodele amphibians, the blastema re‐expresses genes essential for limb development (Gardiner et al. 2002; Endo et al. 2004; Stoick‐Cooper et al. 2007; Nacu & Tanaka 2011). In contrast, the limb blastema of frogs does not show the proper expression of these genes, and presumably for this reason the blastema cannot recapitulate the limb developmental program in frogs (Endo et al. 2000; Yakushiji et al. 2007; Ohgo et al. 2010). Therefore, many attempts have been made to enhance the limb regenerative ability in frogs by applying knowledge about limb development. In particular, the ability of functional joints to regenerate can be regarded as an important key step to bridging the gap between regenerative and non‐regenerative species.

Analysis of limb development using various vertebrates including chicken and rat revealed that the future elbow joint site is first determined at the intersection of Y‐shaped uninterrupted condensed mesenchyme (Hinchliffe & Johnson 1980). While the cells at future joint sites differentiate into interzone cells, the rest of the condensed mesenchymal cells differentiate into chondrocytes, resulting in interruption of the cartilaginous nodule at future joint sites (Holder 1977; Mitrovic 1978). Bone morphogenetic protein (Bmp) family members, growth/differentiation factor 5 (Gdf5) and Bmp4 are important for interzone cell differentiation and joint development in various vertebrates including chicken, mouse, and frog (Storm et al. 1994; Satoh et al. 2005b; Koyama et al. 2008). In a developing limb, the expression of *Gdf5* is first observed in condensing mesenchyme that forms cartilage before joint formation. At later stages, *Gdf5* expression is localized in the perichondrial layer around the cartilage elements and interzone cells. After that, *Gdf5* expression becomes restricted to interzone cells (Storm et al. 1994; Francis‐West et al. 1999; Satoh et al. 2005b).

Loss‐of‐function and gain‐of‐function studies of Gdf5 in vivo and in vitro have highlighted the chondrogenic activity of Gdf5 during development in vertebrates including chicken, mouse, and humans (Storm et al. 1994; Storm & Kingsley 1996, 1999; Thomas et al. 1997; Francis‐West et al. 1999; Merino et al. 1999). However, the descendants of *Gdf5*‐expressing interzone cells give rise not only to articular cartilage but also to non‐cartilaginous joint tissues, including synovial linings and ligaments (Koyama et al. 2008). The anti‐chondrogenic activities of Wnt/β‐catenin signaling and noggin are responsible for non‐cartilaginous differentiation of interzone cells and joint morphogenesis in chicken and mouse (Brunet et al. 1998; Hartmann & Tabin 2000; Guo et al. 2004; Später et al. 2006a, 2006c; Koyama et al. 2008).

To make the joint functional as a locomotor component, it is also necessary for muscles to be inserted into skeletal tissues via tendons in the appropriate pattern (Schweitzer et al. 2010). In limb development, tendon progenitor cells are differentiated from limb mesenchyme, which originates from lateral plate mesoderm (Kardon 1998; Pryce et al. 2009). The basic helix−loop−helix transcription factor scleraxis (Scx) is a distinct marker of early tendon progenitor cells and mature tenocytes (Cserjesi et al. 1995; Schweitzer et al. 2001; Satoh et al. 2006). During the course of differentiation from tendon progenitor cells into tenocytes, Scx regulates the expression of tendon‐related genes, including collagen type Iα1 and tenomodulin (Shukunami et al. 2006; Léjard et al. 2007). In mature tendon, tenocytes deposit and become embedded in a large amount of extracellular matrix (ECM), including collagen and small leucine‐rich proteoglycans (Yang et al. 2013).

Based on the strategy of recapitulating the joint developmental mechanism within the blastema, several approaches to joint regeneration in frogs have been reported. Exogenous administration of Bmp4 during regeneration can induce joint‐like interruption in the spike cartilage (Satoh et al. 2005b). It has also been reported that multi‐digit regenerates with joint‐like structures can be formed after limb amputation by transplantation of larval limb progenitor cells with activation of Wnt/β‐catenin signaling and treatment with Sonic hedgehog, Fgf10, and thymosin β4 (Lin et al. 2013). Thus, joint‐like structures can be induced in the spike by artificial treatment, but there has not yet been any report of functional joint regeneration in frogs.

On the other hand, recent insights we gained into successful joint regeneration in newt emphasized that, after amputation at the joint, the interaction between the remaining and the regenerated tissues has a significant effect on the morphogenesis of the regenerated elbow in newt and can result in structural reintegration of the remaining tissues and the regenerated tissues into a functional elbow joint (Tsutsumi et al. 2015). The technique of episcopic fluorescence image capture (EFIC) enables observation of the precise three‐dimensional (3D) morphology of the tissues, including bones, cartilage, muscles, and tendons (Weninger & Mohun 2002; Rosenthal et al. 2004; Tsuchiya & Yamada 2014; Tsutsumi et al. 2015).

This finding in newt prompted us to attempt an alternative approach for achieving joint regeneration in frogs in the present study. In this research, we hypothesized that if the limb is amputated at the elbow joint in frogs, the elbow joint might be regenerated as a result of the interaction between the remaining tissues and the regenerated tissues. Consistent with this, we found that, after amputation at the elbow, frogs could regenerate a functional elbow at the proximal end of the regenerated spike cartilage. Observation using EFIC clearly showed that not only the remaining bone and the regenerated spike cartilage, but also muscles and tendons, were structurally integrated into a functional elbow joint. Our results might open up possibilities for unraveling molecular and cellular mechanisms of joint regeneration using *Xenopus laevis*.

## Results

### Functional regeneration of the elbow joint in *Xenopus laevis*

**Figure 1:**
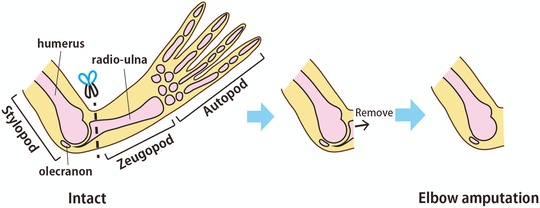
The method of *Xenopus laevis* limb joint amputation. In order to observe spike regeneration from the elbow joint, the forelimb was amputated slightly distal to the elbow joint and the remaining pieces of the radio‐ulna were completely removed with forceps.

In frogs, two skeletal elements in the zeugopod (the radius and ulna) that are separate in many other tetrapods are fused into the radio‐ulna (Fig. 1, left panel). Frogs also have a sesamoid bone, called the olecranon, beyond the proximal end of the radio‐ulna. To investigate the effect of the remaining tissues on the morphogenesis of regenerated tissues in frog, we amputated frogs’ forelimbs slightly distal to the elbow joint and removed the residual amputated radio‐ulna from the stylopod (Fig. 1). After this amputation at the elbow joint, 89.5% (17/19) of frogs regenerated a spike on the stump, a rate comparable to that in frogs whose limb was amputated at the stylopod or the zeugopod level (Tassava 2004).

**Figure 2:**
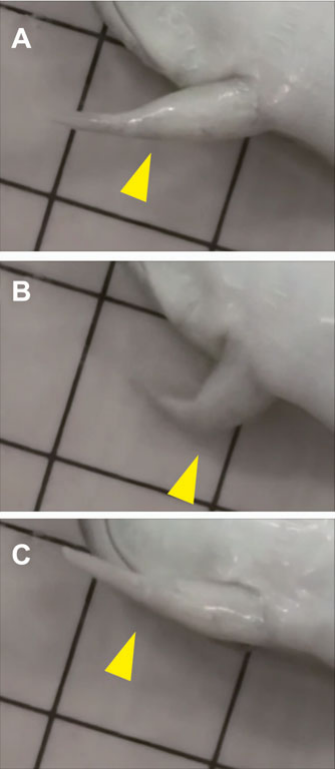
The bending−stretching motion of the regenerated spike (see also Movie S1). After amputation at the elbow, the motion of the regenerated forelimb was observed. (A) The frog stretched the regenerated forelimb. (B) The frog then bent the regenerated forelimb at the elbow joint. (C) The frog then stretched the limb again. Yellow arrowheads indicate the position of the regenerated elbow between the remaining stylopod and the regenerated spike. The sides of the squares in the background are 1 cm.

To examine whether a functional elbow was regenerated between the remaining humerus and the regenerated spike, we observed the movement of these regenerated forelimbs. Interestingly, although the distal side of the original elbow joint was completely eliminated by the surgery, frogs showed bending and stretching motions of their elbow joint in the regenerated forelimb (Fig. 2A−C; Movie S1). This result showed that frogs could regenerate a biomechanically functional elbow joint between the remaining humerus and the regenerated spike. This strongly suggests that the regenerated spike cartilage had a precisely reciprocal joint structure to the opposing proximal part of the elbow joint. Furthermore, since a functional joint requires integration of multiple tissues, including muscles, tendons, and bones, the biomechanical functionality of the regenerated elbow suggested that both flexor and extensor muscles of the stylopod were inserted into the regenerated cartilage via tendons.

### Regeneration of the concave joint morphology after amputation at the elbow

**Figure 3:**
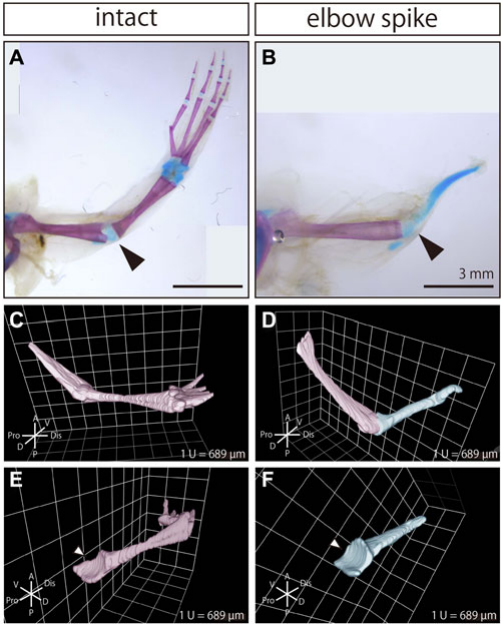
Joint structure was regenerated at the proximal end of the spike. Whole‐mount bone and cartilage staining image of (A) the intact forelimb and (B) the regenerated forelimb after amputation at the elbow. Black arrowheads indicate the position of the elbow joint. Bones were stained magenta, and cartilage was stained blue. 3D reconstructed images of the skeletal tissues in (C) the intact forelimb and (D) the regenerated forelimb after amputation at the elbow, which were obtained with EFIC. 3D reconstructed image showing the skeletal tissues (except for the humerus) in (E) the intact forelimb and (F) the regenerated forelimb (spike). White arrowheads indicate the concave morphology of the elbow joint.

To observe the morphology of the skeletal tissues of the intact and regenerated forelimb, we performed whole‐mount bone and cartilage staining (Fig. 3A, B). The morphology of the remaining elbow joint region of the humerus was almost equivalent to the intact one, and the regenerated cartilage was not continuously connected to the humerus (Fig. 3A, B), which suggested that frogs regenerated concave joint morphology at the proximal end of the spike. To test this possibility, we used EFIC to observe the 3D morphology of the regenerated cartilage. In the EFIC images, the remaining bone was segmented in pink and the regenerated cartilage in blue (Fig. 3C−F), and the 3D reconstructed images showed that the shape of the proximal end of the spike was in reciprocal shape to that of the opposing convex part of the elbow joint in the remaining humerus, which was comparable to the shape of the proximal end of the radio‐ulna in the intact limb (Fig. 3C, D). The concave morphology of the proximal end of the spike was comparable to that of the intact radio‐ulna (Fig. 3E, F).

### Insertion of both flexor and extensor muscles of stylopod into the regenerated spike cartilage

**Figure 4:**
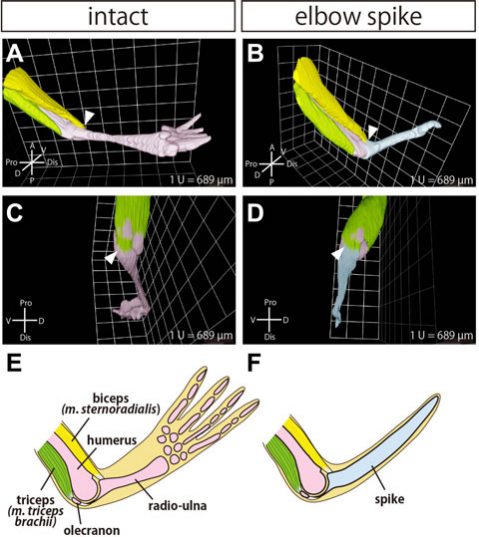
The remaining muscles were inserted into the regenerated spike cartilage. 3D reconstructed image of the skeletal tissues and muscles in (A) the intact forelimb and (B) the regenerated forelimb after amputation at the elbow. The remaining skeletal tissues were segmented in pink, and the regenerated cartilage (spike) was segmented in blue. The biceps (*m. sternoradialis*) and its tendons were segmented in yellow, and the triceps (*m. triceps brachii*) was segmented in green. Arrowheads indicate the insertion site of the biceps. The same reconstructed images are shown from different angles for (C) the intact forelimb and (D) the regenerated forelimb. Arrowheads indicate the insertion site of the triceps. Schematic images of the musculoskeletal structure of (E) the intact forelimb and (F) the regenerated forelimb.

To observe the structure of muscles, tendons, bones, and cartilage in the regenerated forelimb after amputation at the elbow joint, we segmented the muscles and tendons in EFIC images. Among the muscles and tendons which insert into the radio‐ulna in the intact forelimb, we focused on the biceps (*m. sternoradialis*) and its tendons (Fig. 4, yellow), which act as a powerful flexor of the forearm, and the triceps (*m. triceps brachii*) and its tendons (Fig. 4, green), which act as a powerful extensor of the forearm (Ecker 1889). In the regenerated forelimb after amputation at the elbow joint, the distal tendon of the biceps was inserted into the anterior extremity of the regenerated spike, similarly to the insertion of this tendon in the intact forelimb (Fig. 4A, B, arrowheads). Furthermore, the distal tendon of the triceps was inserted into the posterior extremity of the regenerated spike, similarly to the insertion of this tendon in the intact limb (Fig. 4C, D, arrowheads). Thus, the major flexor and extensor muscles which contribute to the bending−stretching motions of the elbow were inserted into the regenerated spike in a similar pattern to those in the intact forelimb (Fig. 4E, F).

### The process of functional joint regeneration after amputation at the elbow

**Figure 5:**
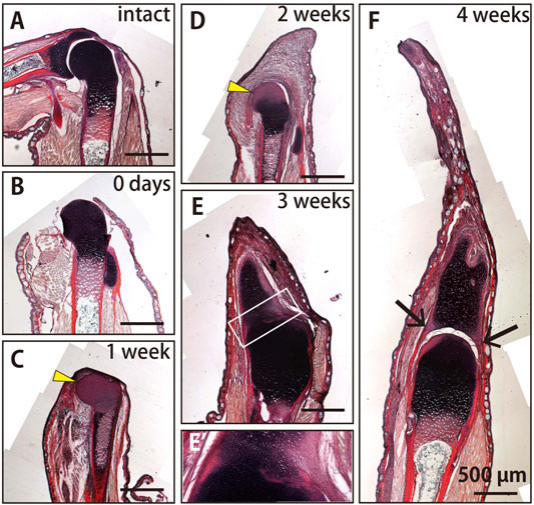
Time course and various tissue components involved in joint regeneration. Histological sections stained by Elastica van Gieson staining of (A) the intact forelimb, (B) the forelimb at 0 days, (C) 1 week, (D) 2 weeks, (E) 3 weeks, and (F) 4 weeks after amputation at the elbow. (E′) A more highly magnified view of the boxed region in (E). Elastic fibers in cartilage were stained purple, collagen fibers in bone and tendons were stained red, muscles were stained yellow, and the cell nucleus was stained black. Yellow arrowheads indicate the loss of the signal for elastic fibers in the remaining joint of the humerus. Black arrows indicate the tendon insertion into spike cartilage.

Next, we examined the time course and various tissue components involved in elbow joint regeneration in frogs after amputation at the elbow by using Elastica van Gieson staining to the sections of regenerating limbs. In this histological staining, elastic fibers in cartilage were stained purple, collagen fibers in bones and tendons were stained red, muscle fibers yellow, and the nucleus of cells black (Fig. 5A). We confirmed that soon after the amputation, the zeugopodal skeletal element was completely eliminated, while the proximal side of the elbow joint remained intact (Fig. 5B). One week after the amputation, wound epithelium covered the stump (Fig. 5C). At 2 weeks after amputation, a blastema was formed between the wound epithelium and the remaining joint cartilage in the humerus, with the shape of this cartilage remaining intact (Fig. 5D). At 1 week and 2 weeks after the amputation, the staining signal for elastic fibers in the remaining elbow joint was lost (Fig. 5C, D, yellow arrowheads), suggesting that the tissue content of the remaining joint was changed. Cartilage could be observed in the blastema at 3 weeks after the amputation (Fig. 5E). Interestingly, we found that the joint cartilage of the remaining humerus and the regenerated spike cartilage were connected at this time (Fig. 5E′). At 4 weeks after the amputation, the elbow joint morphology was regenerated at the proximal end of the spike (Fig. 5F). Furthermore, the tendons of the stylopodal muscles were inserted into the spike cartilage (Fig. 5F, arrows).

**Figure 6:**
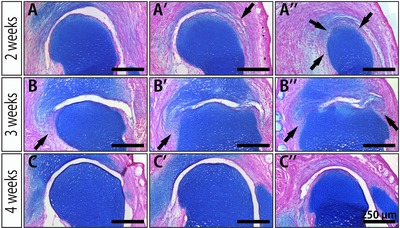
Observation of the reformation of the joint cavity using serial sections. Alcian Blue/Nuclear Fast Red staining of the regenerating joint at (A)−(A″) 2 weeks, (B)−(B″) 3 weeks, and (C)−(C″) 4 weeks after amputation. Each row shows selected serial frontal sections of one particular regenerating joint. Acidic polysaccharides of cartilage were stained blue, and the cell nucleus was stained red. Arrows indicate the connecting part between the remaining articular cartilage and the regenerating tissues.

For more detailed observation of the connection and separation of the remaining articular joint cartilage and the regenerated tissues, we carefully examined serial sections of the regenerating limb at 2 weeks, 3 weeks and 4 weeks after the amputation stained with Alcian Blue/Nuclear Fast Red, which stains acidic polysaccharides of cartilage in blue and the cell nucleus in red (Fig. 6). Because Alcian Blue staining is more sensitive for the detection of cartilage than Elastica van Gieson staining, some staining could already be observed around the remaining articular cartilage at 2 weeks after the amputation (Fig. 6A−A″). At this time, the remaining articular cartilage and the regenerating cartilage were partially connected (Fig. 6A′, A″, arrows). At 3 weeks after the amputation, when the regenerated cartilage was more mature, the remaining articular cartilage and the regenerated cartilage were also partially connected, and the blue signal for cartilage at the connecting part was weaker than that of the mature cartilage (Fig. 6B, B′, B″, arrows). At 4 weeks after the amputation, the connection between the remaining joint cartilage and the regenerated spike cartilage was completely lost (Fig. 6C−C″). These observations suggested that the remaining and the regenerating cartilage were partially connected, and then subsequently separated, to reform the interlocking structure during the process of joint regeneration.

### *Gdf5* expression in the regenerated spike cartilage

It is possible that the partial connection and the subsequent separation of the remaining articular cartilage and the regenerating cartilage in the joint regeneration were equivalent to the interzone cell differentiation in joint development. To examine this possibility, we analyzed the gene expression of *Gdf5* and *Sox9* as markers for joint cells (Koyama et al. 2008) and chondrocytes (Wright et al. 1995; Bi et al. 1999), respectively. First, we checked the reliability of the RNA probes for *Sox9* and *Gdf5* by in situ hybridization of developing frog hindlimbs at stage 54 (Figs. S1A and S1B, respectively). Since the resulting expression patterns were consistent with those previously reported in *Xenopus laevis* and other vertebrates, we concluded that these probes can reliably stain *Sox9‐* and *Gdf5‐*expressing cells by in situ hybridization (Storm et al. 1994; Francis‐West et al. 1999; Satoh et al. 2005b).

**Figure 7:**
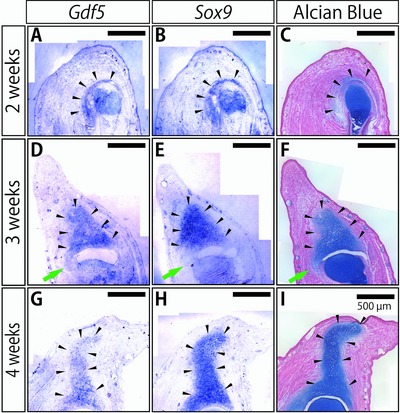
Expression of joint marker genes in the regenerating joints. In situ hybridization of *Gdf5* (a joint cell marker) and *Sox9* (a chondrocyte marker) and Alcian Blue/Nuclear Fast Red staining to serial sections close to each other in the regenerating spike at (A)−(C) 2 weeks, (D)−(F) 3 weeks, and (G)−(I) 4 weeks after the amputation. Arrowheads in each column indicate the signals for the *Gdf5* and *Sox9* expression and the signals for cartilage in Alcian Blue staining, respectively. Green arrowheads indicate the connecting part between the remaining articular cartilage and the regenerating spike cartilage at 3 weeks after the amputation.

Next, we performed in situ hybridization of *Gdf5* and *Sox9* and Alcian Blue/Nuclear Fast Red staining in serial sections during spike formation after amputation at the elbow (Fig. 7). *Gdf5* expression was first observed in the blastema around the remaining articular cartilage at 2 weeks after amputation (Fig. 7A, black arrowheads; showing a serial section very close to that of Fig. 6A″). Although this expression pattern of *Gdf5* seemed to suggest the differentiation of “interzone‐like cells” during the joint regeneration, judging from the overlapping expression of *Sox9* (Fig. 7B, black arrowheads) and Alcian Blue staining (Fig. 7C, black arrowheads), we concluded that *Gdf5* was expressed in the regenerating cartilage. These overlapping patterns of *Gdf5*, *Sox9* expression, and Alcian Blue staining were also observed at 3 weeks (Fig. 7D, E, and F, black arrowheads) and 4 weeks after amputation (Fig. 7G, H, and I, black arrowheads). Moreover, in contrast to the strong expression of *Gdf5* in the interzone during joint development, *Gdf5* was only weakly expressed at the connecting part between the remaining cartilage and regenerating tissues (Fig. 7D, green arrow), together with *Sox9* (Fig. 7E, green arrow) and the Alcian Blue staining (Fig. 7F, green arrow). The fact that *Gdf5* was not specifically expressed in the regenerating joint but rather expressed in the entire regenerating cartilage throughout the regeneration process suggested that the molecular mechanisms of joint regeneration might not be common to those of joint development.

### Wnt/β‐catenin signaling activities in the regenerating joint

It is known that, in addition to the expression of *Gdf5*, Wnt/β‐catenin signaling contributes to the anti‐chondrogenic activities of joint cells during joint development (Brunet et al. 1998; Hartmann & Tabin 2000; Guo et al. 2004; Später et al. 2006a, 2006; Koyama et al. 2008). Thus, we examined whether Wnt/β‐catenin signaling activity is involved in the separation of the partial connection between the remaining articular cartilage and the regenerating tissues. Using the H102 anti‐β‐catenin antibody (Santa Cruz, Santa Cruz, CA), Wnt/β‐catenin signaling activity could be detected as nuclear accumulation of β‐catenin (Fagotto & Brown 2009).

**Figure 8:**
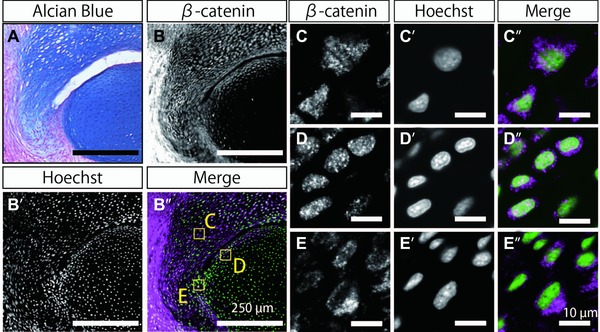
Nuclear accumulation of β‐catenin in the cells of the regenerating joints. (A) Alcian Blue/Nuclear Fast Red staining in the regenerating joint at 3 weeks after amputation (magnified view of Fig. 6B). (B)−(E) Immunostaining with anti‐β‐catenin antibody of a serial section close to that shown in (A). (B′)−(E′) Hoechst staining of the same section as that in (B′). (B″)−(E″) Merged view of immunostaining with anti‐β‐catenin antibody (magenta) and Hoechst staining (green). (C), (C′) The cells in the regenerated spike cartilage in boxed area C in (B″). (D), (D″) The cells on the surface of the remaining articular cartilage in boxed area D in (B″). (E), (E″) The cells in the connecting part between the remaining articular cartilage and the regenerating cartilage in boxed area E in (B″). Bars in (A) and (B)−(B″) indicate 250 μm. Bars in (C)−(C″), (D)−(D″), and (E)−(E″) indicate 10 μm.

At 3 weeks after joint amputation, we could observe the connecting part between the remaining articular cartilage and the regenerating spike cartilage which lacks the Alcian Blue staining (Fig. 8A: more highly magnified view of Fig. 6B). Therefore we expected that if the connecting part was lost during regeneration in a common way with that during joint development, nuclear accumulation of β‐catenin would be observed specifically in the connecting part. The staining of anti‐β‐catenin antibody was detected in almost all cells except for those in the central portion of the remaining joint (Fig. 8B, compared with 8B′). Thus, we observed nuclear accumulation of β‐catenin in the cells in the regenerated spike cartilage (Fig. 8B″, box C), on the surface of the remaining articular cartilage (Fig. 8B″, box D), and in the connecting part between the remaining articular cartilage and the regenerating cartilage (Fig. 8B″, box E). Unexpectedly, although β‐catenin in the nucleus was detected in some of the cells in the regenerated spike cartilage (Fig. 8C−C″) and in the cells on the surface of the remaining articular cartilage (Fig. 8D−D″), we could not detect β‐catenin in the nucleus in the cells of the connecting part (Fig. 8E−E″). Hence, like the expression pattern of *Gdf5*, the pattern of the nuclear accumulation of β‐catenin did not support the commonality of the molecular mechanisms between the interzone cell differentiation during joint development and the separation of the connecting part during joint regeneration.

### Tendon regeneration in the spike and insertion into spike cartilage

The fact that tendons which were associated with the remaining muscles were inserted into spike cartilage (see Fig. 4B, D) strongly suggested that tendons and tendon/cartilage junctions were regenerated in the spike after amputation at the elbow. To observe the process of regeneration of tendons and tendon/cartilage junctions, we examined the expression of *Scx*, which is important for tendon development. First, we confirmed the fidelity of the RNA probe for *Scx* by in situ hybridization of the developing hindlimbs at stage 54 (Fig. S1C, compared with S1D). The signal for *Scx* expression could be detected in developing tendons and in a part of the cartilage in the developing hindlimbs, which is consistent with previous reports in *Xenopus laevis* and other vertebrates (Schweitzer et al. 2001; Satoh et al. 2006).

**Figure 9:**
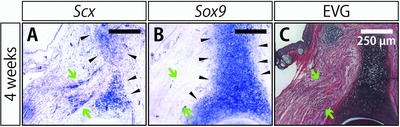
Expression of a tendon marker gene in the regenerating spike. (A) In situ hybridization of *Scx*, a tendon progenitor cell/tenocyte marker. (B) In situ hybridization of *Sox9*, as a chondrocyte marker, in a serial section close to that shown in (A). *Sox9* expression was observed in the regenerating spike cartilage. (C) Elastica van Gieson staining (EVG) of a serial section close to those shown in (A) and (B). Elastic fibers in cartilage were stained purple, collagen fibers in bone and tendons were stained red, muscles were stained yellow, and the cell nucleus was stained black. *Scx* and *Sox9* expressions were observed in the regenerated spike cartilage (black arrowheads) and *Scx* expression was also observed in the extremities of the remaining muscles (green arrows).

Next, we observed the expression patterns of *Scx* and *Sox9* in the regenerating spike at 4 weeks after amputation at the joint by in situ hybridization of serial sections (Fig. 9A, B). The tissue types were identified by Elastica van Gieson staining in a serial section (Fig. 9C). *Scx* expression was observed in the regenerated spike cartilage, largely overlapping with the *Sox9*‐expressing tissues in the regenerated part (Fig. 9A, B, black arrowheads). Some *Scx*‐expressing cells were also observed outside the *Sox9*‐positive region (Fig. 9A, B, green arrows). Comparison with the pattern of Elastica van Gieson staining revealed that these *Scx* single positive cells were located between the remaining muscle and the regenerated spike cartilage (Fig. 9A, C, green arrows), suggesting that tendon progenitor cells were regenerated and connected the extremity of the remaining muscles and tendons to the regenerated spike cartilage.

## Discussion

This is the first report of the regeneration of a functional joint that could show bending−stretching motions in *Xenopus laevis* (Fig. 2). EFIC analysis clearly showed that an elbow structure was regenerated at the proximal end of the regenerated spike cartilage (Fig. 3). Furthermore, at least the tendons anchoring the major flexor and extensor muscles of the remaining stylopod were inserted into the regenerated spike in a functional manner (Fig. 4). These results suggested that the interaction between the remaining and the regenerated tissues composing the joint has the ability not only to determine the joint morphology of the regenerated cartilage but also to reintegrate multiple tissues so as to enable joint functionality.

### Involvement of the ECM recomposition in the remaining joint cartilage during joint regeneration

The process of joint regeneration in frogs observed here has some implications regarding the cellular mechanisms involved. As previously observed in newt joint regeneration (Tsutsumi et al. 2015), the staining of elastic fibers in the remaining joint cartilage was lost during joint regeneration in frogs (Fig. 5C, D). The ECM composition determines various features of the articular cartilage, including mechanical properties of the cartilage and cellular properties of the chondrocytes, by regulating the localization of signaling molecules and transducing integrin‐mediated signals via ECM−cell interaction (García‐Carvajal et al. 2013; Loeser 2014). Thus, it is possible that the observed change in ECM composition might have caused the release of some signaling molecules that stimulated blastema cells and differentiating cells in the regenerating tissues to form a functional joint. Also, it is possible that the chondrocytes in the remaining articular cartilage underwent dedifferentiation and cell proliferation, and these cells contributed to the regenerating spike cartilage via alteration of cellular properties accompanied by the loss of ECM components.

### Reformation of the interlocking skeletal structure

Interzone cell differentiation is a critical step for joint morphogenesis during development (Holder 1977; Mitrovic 1978). Likewise, during limb regeneration in axolotl, interzone‐like cells are involved in joint formation (Lee & Gardiner 2012). Furthermore, excision of bone in the middle of the diaphysis and transplantation of interzone tissue into the excised area causes ectopic joint formation in axolotl (Cosden‐Decker et al. 2012). In this study, we observed that the remaining articular cartilage and the regenerating spike cartilage were partially connected at an early stage of regeneration and then became completely separated by the joint cavity at a later stage. Furthermore, the connecting part showed weak staining with Alcian Blue, suggesting the existence of “interzone‐like” cells (Fig. 6).

**Figure 10:**
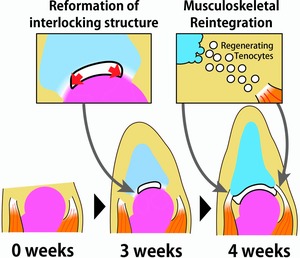
The process of joint regeneration in frogs. Left panel: Amputated frog forelimb after amputation at the elbow joint. Middle panel: At 3 weeks after amputation, the partial connection between the remaining joint cartilage and the regenerating spike cartilage is divided by a cavity between these apposing cartilages, and the interlocking structure is thereby reformed. Right panel: At 4 weeks after amputation, tenocytes are regenerated between the extremity of the remaining muscles and the regenerating cartilage.

However, our results showed that *Gdf5* was not specifically expressed at the connecting part, but rather was broadly expressed in the regenerating spike cartilage, overlapping with the *Sox9* expression and the Alcian Blue staining (Fig. 7). Moreover, we did not observe specific activation of Wnt/β‐catenin signaling in the connecting part; rather Wnt/β‐catenin signaling activity was observed in the cells of the remaining articular cartilage and the regenerating spike cartilage (Fig. 8). Therefore, the molecular analysis did not support the involvement of “interzone‐like” cell differentiation during the joint regeneration. Although the remaining articular cartilage and the regenerating spike cartilage were partially connected, the discontinuity of these tissues seemed to persist around the remaining joint throughout the process of joint regeneration (Figs. 5E′ and 6). Thus, it is possible that this discontinuity might be important for the cells in the connecting part to cease differentiating into chondrocytes and subsequently make the complete separation of the regenerated joint (Fig. 10, middle panel). It is expected that further analysis of joint regeneration will explain the cellular and molecular mechanisms of reformation of interlocking skeletal structures and shed light on new principles of joint formation.

### Mechanisms of musculoskeletal reintegration during frog joint regeneration

Studies of development have revealed that functional integration of multiple tissues of joints is based on developmental interdependence of musculoskeletal tissues. Chondrocytes and tenocytes in limbs share a common origin, lateral plate mesoderm (Kardon 1998; Pryce et al. 2009). The initial onset of *Scx* expression is observed in dorsal and ventral sub‐ectodermal patches (Schweitzer et al. 2001; Satoh et al. 2006; Murchison et al. 2007). In chicken, FGF/MAPK signaling from ectoderm induces *Scx* expression in limb mesenchyme (Schweitzer et al. 2001). Recruitment and aggregation of tendon progenitor cells are regulated by TGF‐β and/or Fgf signaling from muscles and cartilage (Edom‐Vovard et al. 2002; Eloy‐Trinquet et al. 2009; Pryce et al. 2009; Havis et al. 2014). As a result, tendon differentiation proceeds specifically between muscles and cartilage. Gdf5 is also a regulator of tendon development (Wolfman et al. 1997; Mikic et al. 2001). Thus, tendon development is largely dependent on muscle and cartilage. Similarly, in axolotl limb regeneration, muscles are necessary for tendon regeneration in the stylopod and zeugopod, although tendons can be regenerated without muscles in the autopod (Holder 1989).

In frogs, after amputation at the zeugopod, tendon progenitor cells start to differentiate from mesenchymal cells in the regenerating spike, but cannot be matured into tenocytes (Satoh et al. 2006). In contrast, after amputation at the joint, probably because progenitor cells were present between the remaining tendons anchoring the remaining muscles and the regenerated spike cartilage, these cells could be organized into mature tendon tissues (Figs. 5F, 9 and 10, right panel).

Our results also showed that the regenerated spike cartilage expressed both *Sox9* and *Scx* (Fig. 9). The mechanism of tendon/cartilage junction formation during development has been reported to have the following features: whereas the central portion of cartilage is derived from *Sox9* single positive cells, the tendon/cartilage junction is derived from *Sox9*/*Scx* double positive cells, which have multipotency to give rise to chondrocytes and tenocytes (Blitz et al. 2013; Sugimoto et al. 2013), and *Scx* single positive cells intercalate the gap between differentiated tendon and cartilage with tendon (Huang et al. 2013). Therefore, it seems possible that in frog joint regeneration *Sox9*/*Scx* double positive spike cartilaginous cells could give rise to the tendon/cartilage junction.

### Role of mechanical control in frog joint regeneration

Although little is known about how the convex−concave skeletal morphology of the joint is formed, it has been suggested that chondrogenesis and cell proliferation on both apposed sides of the joint are involved in reciprocal joint morphogenesis (Pacifici et al. 2005). Not only skeletal elements but also muscle contraction might play important roles in joint morphogenesis. In mouse embryos, muscle contraction is responsible for joint progenitor cell fate, and without muscle contraction interzone cells differentiate into chondrocytes, resulting in joint fusion (Kahn et al. 2009). Furthermore, paralysis in chicken embryos causes abnormal morphology of knee joints as a result of a local change in cell proliferation (Roddy et al. 2011). Thus, it is plausible that, in the present study, once the remaining muscle inserted into the regenerated spike cartilage, the mechanical force produced by contraction of the remaining muscles also contributed to the interlocking joint morphogenesis at the proximal end of the spike. Thus, the role of muscle contraction in joint regeneration is an intriguing issue that could be amenable to examination using our *Xenopus* joint regeneration system. Paralysis of muscles after blastema formation may give us important insights into the involvement of mechanical force in joint regeneration.

### Future prospects

Our previous study using a urodele amphibian model have revealed that after amputation at the elbow joint a functional joint was regenerated by the mechanism of reintegration based on tissue interactions of the remaining and the regenerated tissues (Tsutsumi et al. 2015). In the present study, we found that even in frogs a functional joint could be regenerated after amputation at the elbow via the mechanism of reintegration. In accord with previous reports (Egawa et al. 2014; Makanae et al. 2014; Mitogawa et al. 2014), our study supports the notion that, compared to the epimorphic and autonomous regenerative system that is highly active in urodele amphibian limb regeneration, reintegrative mechanisms might be more generally used among species. Therefore, the comparative analysis using urodele and anuran amphibian models might provide essential information about the cellular and molecular basis for the mechanism of reintegration. Recently, both urodele and anuran amphibians are increasingly useful model organisms for many genetic approaches, including live imaging, lineage tracing using the Cre‐loxP system, inducible gene expression using the heat‐shock promoter or tet‐on system, and genome editing using the TALEN or CRISPR system (Rankin et al. 2011; Yokoyama et al. 2011; Ishibashi et al. 2012; Takagi et al. 2013; Hayashi S et al. 2014; Hayashi T et al. 2014; Nakayama et al. 2014; Hayashi & Takeuchi 2015). Furthermore, considering that the mechanism of reintegration might be broadly used, we expect that greater understanding of the mechanisms of reintegration achieved using amphibian models might open up a new avenue with the potential for achieving functional joint regeneration in mammals.

## Materials and methods

### Animals

*Xenopus laevis* froglets and tadpoles were offspring of sexually mature adult animals that were obtained from the *Xenopus*‐inbred strain resource center (Hyogo, Japan). The animals were kept in plastic containers in dechlorinated tap water at 26°C and fed daily. All animals were maintained and manipulated according to a protocol approved by the Animal Care and Use Committee of Kyoto University.

### Forelimb amputation

Before surgery, froglets were anesthetized by addition of 0.2% ethyl 3‐aminobenzoate methanesulfonate (Sigma‐Aldrich Co., St Louis, MO) to the water surrounding them. After checking that the animals were well anesthetized, the forelimb was amputated slightly distal to the elbow joint, and the amputated radio‐ulna was removed with forceps so that the amputated surface became flat and the humerus did not degenerate during regeneration (Fig. 1).

### Whole‐mount bone and cartilage staining

For whole‐mount bone and cartilage staining, the forelimbs were fixed with 70% ethanol for 1 h and 100% ethanol for 1 h. Cartilage was stained with 0.002% Alcian Blue 8GX (Sigma‐Aldrich Co.)/20% acetic acid/80% ethanol for 3 days and washed with 100% ethanol for 4−5 days. Tissues were cleared with 0.3% KOH for 1 h and pigments were decolorized with 0.1% H_2_O_2_ overnight. Bones were stained with 0.005% Alizarin Red S (Sigma‐Aldrich Co.)/0.3% KOH. The samples were further cleared with glycerol. Images of samples were obtained with a stereomicroscope Leica M125 (Leica Microsystems, Wetzlar, Germany).

### EFIC imaging and 3D reconstruction

Sample preparation and EFIC imaging were performed as previously described, with some modifications (Weninger & Mohun 2002; Rosenthal et al. 2004; Yamada et al. 2010; Takaishi et al. 2014; Tsuchiya & Yamada 2014; Tsutsumi et al. 2015). Briefly, the forelimbs were fixed with 4% paraformaldehyde/10% methanol/70% phosphate‐buffered saline (PBS) at 4°C overnight and decalcified with 22.5% formic acid/10% sodium citrate/70% PBS for 2 days. Then the samples were dehydrated with ethanol and xylene and embedded in 25% Vyber/4.4% stearic acid/0.4% Sudan IV/70% paraffin. The blocks were serially sectioned at 10‐μm thickness using a Leica SM2500 sliding microtome (Leica Microsystems). The autofluorescent signals of tissues exposed on the block surface were detected with a Hamamatsu ORCA‐ER low‐light CCD camera (Hamamatsu Photonics K.K., Shizuoka, Japan).

The monochrome tissue images obtained with EFIC were modified and segmented using Adobe Photoshop (Adobe Systems Inc., San Jose, CA). Tissue types were identified by referring to Elastica van Gieson stained tissue images (Fig. S2). The segmented sets of 2D tissue images were reconstructed into 3D images using Volocity (Improvison/Perkin Elmer, Waltham, MA).

### Histological staining

Stage 54 tadpoles (Nieuwkoop and Faber 1994) and the regenerated forelimbs of froglets were fixed with MEMFA at room temperature, immersed in 10% sucrose/70% PBS and 20% sucrose/70% PBS, and embedded in O.C.T. compound (Sakura Finetek Japan, Tokyo, Japan). The blocks were sectioned at 10‐μm thickness. The sections were stained with Elastica van Gieson staining and Alcian Blue/Nuclear Fast Red staining. The images were obtained using an upright microscope BX62 (Olympus, Tokyo, Japan) and CCD camera CoolSNAP fx (Photometrics, Tucson, AZ).

### Probe preparation

Partial cDNAs of *Xenopus laevis Sox9*, *Gdf5*, *Scx* were obtained by reverse transcription polymerase chain reaction (PCR) using a total cDNA sample obtained from the whole body of a *Xenopus laevis* tadpole as a template. Primers used for *Xenopus laevis* were *Sox9b* (forward primer, 5′‐CAAGACGCTGGGGAAGTTATGGA‐3′; reverse primer, 5′‐GGATTGATGGAACTCCCGTTGTG‐3′; NM_001094473.1), *Gdf5* (forward primer, 5′‐CTTTGACATCAGTGCTTTGG‐3′; reverse primer, 5′‐TTCTTATTAGGCCTCTTCCC‐3′; NM_001092997.1, Satoh et al. 2005b) and *Scx* (forward primer, 5′‐ TGTCTGATGAGGAGGAGGAGGAGA‐3′; reverse primer, 5′‐ CTGGTTGCTGAGGCAGAAGGTG‐3′; NM_001098682.1). The PCR products were cloned using a TOPO TA Cloning Kit (Life Technologies, Carlsbad, CA). Digoxigenin (DIG) labeled anti‐sense and sense RNA probes were synthesized using T7 RNA Polymerase (Life Technologies) and Sp6 RNA Polymerase (Promega, Madison, WI).

### In situ hybridization

In situ hybridization was performed as previously reported (Ohgo et al. 2010) with some modifications. Briefly, the cryosections were treated with 5 μg/mL proteinase K at 37°C for 8 min. After re‐fixation with 4% paraformaldehyde in PBS for 20 min, each DIG labeled RNA probe was diluted in hybridization buffer [50% formamide, 5× saline sodium citrate (pH 5.0), 10 μg/mL yeast tRNA, 0.1% CHAPS (3‐[(3‐cholamidopropyl)dimethylammonio]‐1‐1 propanesulfonic acid), 100 ng/mL heparin, 1× Denhardt's solution, 10 mmol/L ethylenediaminetetraacetic acid (pH 8.0), 0.1% Tween 20] and were hybridized at 58°C overnight. The DIG of the hybridized probe was immunoreacted with anti‐DIG‐AP antibody (1/2000) (Roche Applied Science, Penzberg, Germany). The sections were incubated with 200 μg/mL NBT (Roche Applied Science) and 175 μg/mL BCIP (Roche Applied Science) in NTMT (100 mM NaCl, 100 mM Tris‐HCl (pH9.5), 50 mM MgCl_2_, 1% Tween 20). The signals were detected using a BX62 upright microscope (Olympus) and CCD camera CoolSNAP fx (Photometrics).

### Immunofluorescence

The cryosections were rinsed with Tris PBS (TPBS) three times for 5 min each, post‐fixed with 4% paraformaldehyde in 70% PBS for 30 min at 4°C, and blocked with 1% blocking reagent (Roche Applied Science). Then the sections were incubated with rabbit anti‐β‐catenin antibody (H102, 1/100 dilution, Santa Cruz) overnight at 4°C. The sections were washed five times in TPBS and then the sections were treated with secondary antibody, Alexa594 labeled anti‐rabbit IgG(H+L) antibody (Invitrogen, Carlsbad, CA), for 30 min at room temperature, washed three times in TPBS, and stained with 10 ng/μL Hoechst 33342 (Invitrogen) for 3 h at room temperature. Fluorescence was detected with a BX62 upright microscope (Olympus) and an FV10 confocal microscope (Olympus).

## Supporting information

Additional Supporting Information may be found in the online version of this article at the publisher's website:

**Movie S1**. The bending‐stretching motions of the regenerated forelimb after amputation at the elbow.

**Figure S1**. Fidelity check of RNA probes for Sox9, Gdf5, and Scx.

**Figure S2**. Segmentation of EFIC image.
